# Advances in Osteoporotic Bone Tissue Engineering

**DOI:** 10.3390/jcm10020253

**Published:** 2021-01-12

**Authors:** Cosmin Iulian Codrea, Alexa-Maria Croitoru, Cosmin Constantin Baciu, Alina Melinescu, Denisa Ficai, Victor Fruth, Anton Ficai

**Affiliations:** 1 Department of Science and Engineering of Oxide Materials and Nanomaterials, Faculty of Applied Chemistry and Materials Science, University POLITEHNICA of Bucharest, 060042 Bucharest, Romania; codrea.cosmin@yahoo.com (C.I.C.); croitoru.alexa@yahoo.com (A.-M.C.); anton.ficai@upb.ro (A.F.); 2 Department of Oxide Compounds and Materials Science, Institute of Physical Chemistry “Ilie Murgulescu” of the Romanian Academy, 060021 Bucharest, Romania; vfruth@gmail.com; 3 Anaesthesia Intensive Care Unit (AICU/ATI), Department of Orthopedics, University of Medicine and Pharmacy “Carol Davila”, 020021 Bucharest, Romania; cosminbaciu@hotmail.com; 4 Department of Inorganic Chemistry, Physical Chemistry and Electrochemistry, Faculty of Applied Chemistry and Materials Science, University POLITEHNICA of Bucharest, 060042 Bucharest, Romania; denisaficai@yahoo.ro; 5 Academy of Romanian Scientists, 050094 Bucharest, Romania

**Keywords:** bone, biomaterial scaffolds, 3D printing, osteoporosis, strontium ranelate

## Abstract

The increase in osteoporotic fracture worldwide is urging bone tissue engineering research to find new, improved solutions both for the biomaterials used in designing bone scaffolds and the anti-osteoporotic agents capable of promoting bone regeneration. This review aims to report on the latest advances in biomaterials by discussing the types of biomaterials and their properties, with a special emphasis on polymer-ceramic composites. The use of hydroxyapatite in combination with natural/synthetic polymers can take advantage of each of their components properties and has a great potential in bone tissue engineering, in general. A comparison between the benefits and potential limitations of different scaffold fabrication methods lead to a raised awareness of the challenges research face in dealing with osteoporotic fracture. Advances in 3D printing techniques are providing the ways to manufacture improved, complex, and specialized 3D scaffolds, capable of delivering therapeutic factors directly at the osteoporotic skeletal defect site with predefined rate which is essential in order to optimize the osteointegration/healing rate. Among these factors, strontium has the potential to increase osseointegration, osteogenesis, and healing rate. Strontium ranelate as well as other biological active agents are known to be effective in treating osteoporosis due to both anti-resorptive and anabolic properties but has adverse effects that can be reduced/avoided by local release from biomaterials. In this manner, incorporation of these agents in polymer-ceramic composites bone scaffolds can have significant clinical applications for the recovery of fractured osteoporotic bones limiting or removing the risks associated with systemic administration.

## 1. Introduction

Bone grafting is a common surgical method used to improve bone regeneration in orthopedic practice. In the case of considerable diminution of bone mass, bone grafts are essential because the self-healing process would be slow or the could entirely fail [1]. At an estimated total of 2,000,000 bone graft procedures carried out every year, bone repair frameworks remain a promising line of research because of the continuously growing request for it and the reduced stock of bone substitutes [1,2]. Bone tissue engineering aims at delivering novel methods for treating bone tissue deficiencies often resulting from polytrauma, pathological fractures, and osteonecrosis [2] as there is an increasing need to provide functional replacement grafts for the patients [3].

Progresses in medicine have raised both life expectancy globally and age-connected illnesses liable for declining life quality of the elderly [4], with one of the most common and wide-spread skeletal disease being osteoporosis [5], with an increase in osteoporotic fracture in both genders with age [6]. Each year, almost 200,000,000 patients are confirmed as osteoporotic and around 9,000,000 osteoporotic fractures happen globally [5], with a rising number of elderlies being prone to fragility fractures [7]. Bone lesions are difficult for the elderly and can lead to morbidity and substantial socioeconomic costs creating a demand for new and efficient regenerative strategies and better results for patients [4] as the incidence of osteoporosis is predicted to further rise worldwide [5]. This emphasizes the importance and value of preclinical bone repair frameworks capable to transfer into suitable clinical use even more [2]. 

Bone tissues display inherent regrowth and self-repair features to some extent, but the capacity of the injured bones for the natural process of healing and restore load-bearing function is often insufficient, resulting in fracture nonunion [2]. Larger defects, referred to as critical-size defects, demand clinical interventions in order for the bone to repair or regenerate itself [8]. The ways in which large bone injuries are treated consist of autografts, allografts, and scaffolds [1,4] but currently none of them are adequate because of their specific limitations for instance reduced bioactivity, possible pathogens spreading, inflammation, supplementary surgery, restricted supply, unfit shape and size, and donor site related morbidity [4]. 

This review reports on the latest advances in biomaterials containing biologically active agents used for their ability to enable osteointegration and osteogenesis in osteoporotic skeletal defect sites. The loco-regional delivery is especially useful because they can assure the desired level of these agents at the bone level, to assure a higher bioavailability and thus to minimize the probability of implant rejection and faster osteointegration and healing. Most of these drug delivery systems are designed to be used as grafting materials to restore the normal ratio between resorption and bone formation and to counteract the effects of osteoporosis.

## 2. Bone Tissue

**Figure 1:**
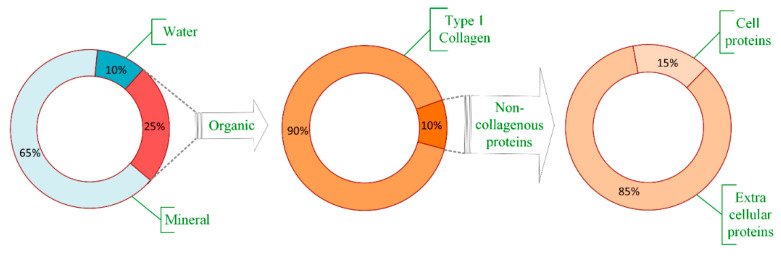
Bone composition. Realized based on [10].

Bones are living organs constantly modeling and remodeling throughout life and serve as reservoir for calcium, phosphate and many other bodily elements, thereby, assuring their homeostasis [9]. Bone consists of an organic matrix, mineral components, and water, as presented in Figure 1. The bulk part of the organic matrix consists of type I collagen, but other non-collagenous proteins are also present, mainly extracellular and a small part within the cells [10]. Bone composition, by weight, is approximately 65% mineral (mostly as hydroxyapatite (85%), but also calcium carbonate and calcium fluoride [9]), organic part is approximately 20% to 25%, mostly type I collagen (about 90%) [10], while the water content varies between 10% and 20% depending on several factors including age, species, bone health, etc. [11]. 

Bone tissue can assume a compact (cortical bone) or trabecular (cancellous bone) structure [12], but indifferently, all experience remodeling throughout life associated with bone resorption, a process performed by osteoclasts subsequent bone formation through the action of osteoblasts [5].

Cortical bone consists of a dense matrix constructed by recurrent osteon units with collagen fibers assembled in a concentric manner around a central canal containing blood vessels [13]. This structure formed by collagen fibers is oriented along with the directions of the loading lines, greatly confirmed with the piezoelectric assistance involved in bone formation [14]. It forms a dense outer shell [9] consisting of well-organized lamellae of hierarchical structures [5] and provides torsion, bending resistance, and compressive strength [9]. They possess remarkable strength but lower capacity to bear loads beyond the elastic deformation range in contrast to trabecular (cancellous) bone, consisting in unparallel fibrillar units of variable porosity (ranging between 50–90%) [5]. 

Cancellous (trabecular) bone is found in the porous interior [9], possesses a network of interlinked trabeculae containing marrow, organized in a hierarchical manner extended between solid material, trabeculae, lamellae, and collagen-hydroxyapatite composite and having disorganized collagen network [13]. The trabeculae exhibit considerable surface area in which nutrients diffusion and growth factors circulation is easily done, allowing, thus, cancellous bone to play a metabolically active role and permit a more persistent remodeling compared to cortical bone [9]. The mechanical capacity of cancellous bone derives to a great extent from its bone mineral density (BMD), while the stiffness of cortical bone derives to a great extent from its porosity [5]. 

Bone tissue is distinct due to its capacity to heal free of scar tissue, which determines an unscarred full restoration of bone tissue integrity [15]. Fractured bone repair itself by repeating various steps of endochondral and intramembranous bone formation and healing is devoid of scar tissue. Development of hematoma implies inflammation at the injured site and the action of signaling molecules involved in regulating new bone development (interleukins, tumor necrosis factor-a, fibroblast growth factors, platelet-derived growth factor, vascular endothelial growth factor (VEGF), bone morphogenetic proteins (BMPs), etc.) [9]. Inflammatory cells (macrophages, monocytes, lymphocytes, and polymorphonuclear cells) and fibroblasts infiltrate bone tissue in a process mediated by prostaglandins [11]. Throughout fracture repair, mesenchymal stem cells (MSCs) from the bone marrow are engage at the fracture site in interaction with the local cells influencing the healing efficacy [7]. The emergence of intramembranous bone starts right away at the cortical tissue and periosteum. Later, the fracture stabilization occurs through the action of the outer soft tissues through callus formation. Afterwards, chondrocyte proliferation happens. Ingrowing of blood vessels starts to bring chondroclasts to the site, which reabsorb calcified cartilage, and osteoblastic progenitors, which launch new bone tissue formation [9]. The recruitment of osteoblast on the surface and the emergence of new bone tissue are moreover promoted by the modifications in the ionic dynamics of the microenvironment [16]. Mechanical continuity of the cortical bone tissue is reached through later remodeling of recently formed bone. If the needed regeneration of bone starts due to damage or disease, hematoma building and a quick inflammatory response occurs as a way to facilitate host cell attraction and the discharge of decisive signaling molecules [9]. Hematoma building and inflammation process starts bone healing, which further elapses via the major stages of anti-inflammatory signaling, revascularization, soft callus development, its mineralization and remodeling in the end, macrophages playing an essential role in the process [15], influencing, thus, the success or failure of a bone implant [17]. Macrophages control cell migration to the injured site through the cytokines and growth factors they produce (tumor necrosis factor-a, IL-1, IL-6, IL-8, IL-12, TGF-b, platelet-derived growth factor, and insulin-like growth factor-1), and have effect on cell proliferation, collagen synthesis and angiogenesis [18]. Monocytes, neutrophils, and natural killer cells are also present in the affected site [19]. Inflammatory markers are the following cytokines: TNF-α, IL-1β and iNOS [20]. Osteogenesis, osteoclastogenesis, and angiogenesis are greatly regulated by immune cells and their signaling molecules [21].

Initial inflammatory action is necessary to start the bone healing process but persistent inflammatory action against the implant usually leads to granulation (formation of connective tissue) and formation of a fibrotic capsule around the implant with undesirable results [20] or increased healing time [22].

Bone tissue extracellular matrix is formed out of a non-mineralized organic constituent, largely type-1 collagen with numerous post-translational modifications, but also non-collagenous proteins particularly involved in improved fusion between the mineral crystals and the organic matter [5], and a mineralized inorganic constituent (made up of carbonated apatite mineral particles in the form of 4-nm-thick plates) [8,12]. The nano-composite structure (collagen fibers reinforced by hydroxyapatite) is necessary for compressive strength and toughness towards fracture [12], thus, bearing an impact without cracking [5]. The toughness and flexibility of the collagen fibers adjust bone ductility. 

The metaphyseal component of bone is largely made up of cancellous tissue, which is metabolically more active compared to cortical tissue. As a consequence of this attribute, osteoporosis can impact its mass and structural integrity at a higher rate [23], mainly because diffusion is much higher as surface to volume ratio is superior in contrast to the cortical one. 

Healing rates are age dependent, as in young people fractures usually heal about the weight-bearing level in approximately six weeks, and about complete mechanical integrity level after approximately one year, and in the elderly individuals the healing rates can be much lower [24]. In pathological or large fractures and defects, the processes of healing and repair can be unsuccessful, due to deficient blood delivery, bone cells or bone forming minerals, but also due to infection in the bone or its adjacent tissues, and systemic affections causing delay or even lack of union [25]. Important factors that affect the process of bone tissue healing are also mechanical stability, proportions of the affected site, severity and incidence of adjacent tissue injuries [11]. 

After implantation of bone scaffolds in the body, injured blood vessels quickly lead to protein adsorption on scaffold surface and formation of fibrin-rich clot with the role of a transitory surface matrix at the tissue-scaffold interface occurs. It emits various pro-inflammatory cytokines and chemokines, triggering the mobilization of inflammatory and osteoprogenitor cells towards the infixed scaffold. The scaffold is recognized as a foreign body by the immune system, inducing specific immune reactions. The scaffold active regulates the kind and extent of immune system reactions during bone regeneration [17]. To counteract chronic inflammatory action against implants, anti-inflammatory factors such as corticosteroids and prostaglandins were investigated but adverse effects such as hepatotoxicity, cardiotoxicity, and immunological impairment in long-term use were reported. Extended inflammation may cause fibrosis, granuloma formation and further encapsulation and failure of the implant [20].

## 3. Bone Healing and Types of Bone Grafts

Bone grafts are made of biomaterial implanted in order to assist healing alone or with the aid of other materials, because of the expected properties of osteogenesis, osteoinduction, and osteoconduction either alone or combined [25]. Bone grafts are especially needed if a larger bone lost is recorded. It is worth to mention that in fact, only the need of blood is higher than the need of bone grafts. It is also important to mention that synthetic bone grafts have some advantages against autografts, and this is why many synthetic grafts are studied worldwide [26]. 

Autografts are obtained by taking bone from another part of the patient’s own body [11] and still serves as a point of reference to which other grafts may be compared [1,8,11,27] because of their histocompatibility, absence of immunogenic reactions and all other properties demanded of a bone graft material, respectively osteoinduction (growth factors to drive the regeneration process, i.e., bone morphogenetic proteins), osteogenesis (i.e., osteoprogenitor cells, functionally active osteoblastic cells to produce new bone matrix) and osteoconduction (i.e., three-dimensional, porous matrix) [2,12]. Autografts integrate into the host bone faster [1] and are the most effective method for bone regeneration [11], unfortunately donor site morbidity is a problematic consideration [3,8]. Important health risks such as major vessel or visceral injuries during harvesting are also among the limitations in using autografts [25].

Allografts consist of taking bone from the donor and has a lower rate of incorporation within the bone [11]. They are linked to risks of immune reactions and infection carrying, have low osteoinductive capacity and have no bone cells, since donor grafts are devitalized and processed through irradiation or freeze-drying [12]. The handicap of restricted donor supply and the need for immunosuppressive therapy [3] and batch to batch variation [28] add to the ones previously mentioned. 

Xenografts are derived from species other than the recipient, are osteoconductive, relatively inexpensive, do not lengthen the healing time, and the need for a second surgical site for bone harvesting is eliminated [29]. They carry a rare risk of transmission of zoonotic diseases [25], causing an immune reaction, and are unable to gain adequate height and width for large defects [29]. Remains of bovine bone from xenografts can still be unresorbed even after three years as some histological analyses have proved. They are osteoconductive rather than osteoinductive [30] and are considered to be more liable to rejection, even in an aggressive manner [25]. 

Biomaterial scaffolds (engineered scaffolds) address the limits of autografts, allografts and xenografts. Novel approaches offered by tissue engineering have been conducted with the aim to create grafts for repairing and regenerating damaged tissues [4] through the use of a provisional biomaterial scaffold at the injured site in order to stimulate healing and ensure certain recovery of functionality [3] and harness bone innate regenerative capacity, as bone has a natural potential to repair, remodel, and regenerate itself [24]. 

Scaffolds need to be engineered with features that provide cells with signals for regeneration in order to cancel the need for extended in vitro culture anterior to implantation. Hence, considerable efforts are committed to advance biomimetic biomaterials to a more complex stage, making them able to incorporate multi-functionality and possess bioinstructive and stimuli-responsive properties [24].

Scaffolds should possess cytocompatibility and imitate biochemical and mechanical properties of the veritable bone, furthering similar biological functions in order to prevail limitations such as donor deficit, immunogenic reaction, and infection carrying, with the main strategies considering the admixture of cells, biologically active molecules, and impermanent 3D fine-tuned porous design [4]. Degradation capability is necessary to integrate the scaffold and should progress at a rate capable of maintaining mechanical support in implantation sites. Apart of that, it plays a critical role in not only in ensuring the means for metabolite diffusion and the development of new blood vessels, but also in releasing the agents loaded into the biomaterial [3]. Scaffolds enable osteogenic cells adhesion of and provide a suitable microenvironment for them in order to secrete osteoid, the matrix of recently formed tissue and deliver signaling molecules to the bone regeneration space (osteoconduction) [2].

## 4. Biomaterial Scaffolds

Scaffold development usually starts by choosing the scaffold material [3] as chemical composition of the biomaterial plays an essential part in the favorable outcome of the process [9,12]. We distinguish three classes of scaffolds in line with their material composition: metals, polymers, and bio-ceramics, but they may consist also of a combination of these three material types as composites [2,9,27]. Common materials used and approved, such as stainless steel, poly(lactic-co-glycolic acid), and hydroxyapatite can be appealing as they are already safely used as components in existing products [3]. However, they still have their disadvantages such as the necessity for several surgical intervention, stress shielding, wear particle osteolysis [28] and no potential for drug delivery in the case of biocompatible metals and their alloys [16] and inferior mechanical properties that limit its use in load bearing bone parts in the case of bioactive glasses [28]. A further classification is made by considering the origin of the material (natural or synthetic) and its degradation potential under physiologic conditions (resorbable versus non-resorbable) [2]. 

Other characteristics to take into account is the heterogeneousness of the scaffold, that bone is biologically and biomechanically variable, the ease to handle it as a one-step procedure, its ability to reach suitable mechanical properties in order to bear the load of normal movements, and its ability to allow vascularization in order to obtain a normal bone structure [11], which can thus prevent the scaffold to undergo ischemia and cell death [25]. 

Scaffold material aims to support the viability of the adequate cell category and simultaneously operate as a nonpermanent replacement of the bone extracellular matrix, as a substitute matrix that will preferably be displaced by newly functional bone tissue [31], while reproducing the properties of normal bone tissue formation [9]. Scaffolds designed to aid regeneration need to encourage migration, cell adhesion, proliferation, and differentiation [8] and ideally some essential features and functions should be certainly considered: architecture, cytocompatibility, bioactivity, biodegradability, and mechanical characteristics [9].

Material characteristics, such as biomaterial porosity and its surface properties, for instance nano/micro topography, play a decisive role in osteoinduction [9,12] and the expected histogenesis [31]. The architectural features consider a controlled porous microstructure and high porous interconnectivity, a controlled degradation rate, mechanical stability, and osteoconductive properties [8]. In addition, for an effective interaction between scaffolds and the cellular component, other design parameters are necessarily acknowledged, such as surface properties, permeability, geometric properties, and mechanical strength, all of which influence nutrients and oxygen transport throughout the scaffolds [32] and influence cell-material interactions [33] controlling, thus, bone regeneration. 

**Figure 2:**
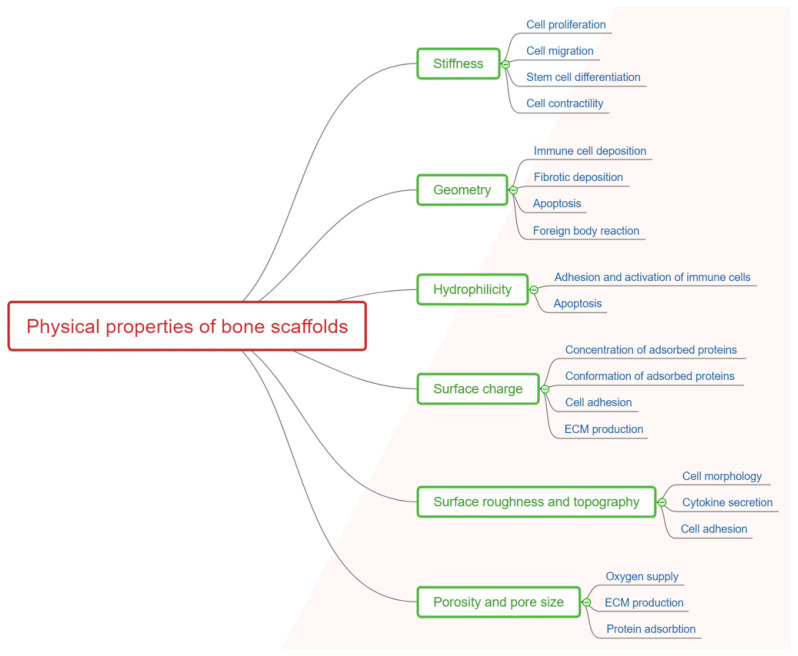
Physical properties of bone scaffolds and their effects on host and bone regeneration. Realized based on [17].

Host reactions to the implanted scaffold are greatly influenced by the physical (Figure 2) and chemical properties of the scaffold and design strategies should consider and evaluate this impact [17]. 

The ability of cells to penetrate, proliferate and differentiate are greatly influenced by pore size, distribution and scaffold geometry, and also the rate of scaffold degradation [34].

Porosity: interconnection between pores promotes the loading of cells into biomaterials due to large internal surface area, which ensures attachment and spreading sites. Because the biomaterial is designed as an open network structure, diffusion of oxygen, metabolites, and growth factors is possible, thus, enabling cells viability and proliferation, and the space needed for the deposition of proteins secreted by the cells. Porosity of the bone tissue facilitate the penetration of host cells and the growth of blood vessels that provide the means to feed the emerging tissue [31]. This microstructure permits the ingrowth of cells, which lead to tissue regeneration. Uniform cell seeding and nutrient exchange are dependent on the interconnectivity of pores [8]. By raising the available surface inside a pore to which cells can adhere on the implant, known as the specific surface area (SSA), a quicker growth of hydroxyapatite appears and thus bone bonding will be more quickly as well [35]. 

Scaffold architecture needs to consider, in order to assure well-adjusted biological and physical properties of scaffolds, the following key parameters: total porosity, pore morphology, distribution, and size [34]. Given the size of the bone cells of about 20 to 35 µm, materials with a porosity of about 50 to 150 µm seem to be the optimal for bone grafting because, in this case, the bone cells can easily penetrate inside, and can assure an in depth osteointegration of these synthetic grafts. If the pore size increases, it means that the mechanical properties decrease and also the time requested for the feeling of these pores with new bone increase, which overall means a slower healing and a higher risk of secondary failure [36,37,38].

Interaction between cells and scaffolds is done mostly through the chemical groups (ligands) present on the biomaterial surface. Using materials found naturally in the extracellular matrix (e.g., collagen) have the advantage of initially possessing ligands in the scaffold shaped as Arg-Gly-Asp (RGD) binding sequences, in contrast with synthetic materials, which may need a planned addition of ligands via, for example, protein adsorption. Ligand density is influenced by the specific surface area which is conditional on the mean pore size in the implanted scaffold. Cells migrate into the scaffold if the pores are sufficiently large for them to penetrate, but pores need to be sufficiently small to reach a satisfactory specific surface, with a ligand density high enough to permit adherence of a substantial number of cells to ligands within the scaffold [24].

Macroporous scaffolds (ranging between 100 and 600 μm) permit superior integration in the host bone, further angiogenesis, and bone distribution. A higher pore size determines an enhancement in permeability, which leads to better bone ingrowth, while small pores have better results for soft tissue ingrowth [34]. For tissue ingrowth, a minimum pore size about 50 µm is necessary [4] while, in most cases, surpassing a pore size limit of 150 µm determines the abrupt decrease of mechanical properties. It is also important to mention that pore size and porosity play an important role in drug delivery, as will be later discussed. Porosity can influence the scaffolds’ ability to induce new blood vessel formation [19]. 

To create pores or increase porosity of biomaterials used in scaffolds, the following methods are used: freeze-drying, salt leaching, gas foaming, controlled air drying, the use of additional polymers, electrospinning, and inclusion of degradable hydrogel microparticles [31,39,40]. Research is still ongoing regarding whether uniform pore distribution and similar size is a better choice than scaffolds designed with irregular pore size distribution [34]. In 3D printing, two kind of pores can be developed, one induced by printing larger pores, which are larger than the intrinsic porosity of the strands and are essential in a faster cell penetration deep inside the graft. The strands can also have pores suitable for the penetration of the osteoblasts or, at least, to assure the attachment of these cells on the scaffold [41].

Degradation: chronic foreign body responses generate complications for non-degradable biomaterials, so the ideal scaffold is ultimately replaced by new native bone tissues [31] as scaffolds are not intended to be perpetual implants [32]. 

Degradation rates depend not just on the chemical composition, but also on pore morphology, although this factor is less significant but should still receive attention in some cases [42]. The degradation rate of the implanted construct is an essential factor in its success. Material degradation should be in accordance to tissue synthesis to ensure appropriate mechanical stability in the course of bone tissue hystogenesis. The required residence time of the scaffold is specific to every tissue and need to be sufficient for cells to populate the scaffold appropriately and produce an enduring bone extracellular matrix. If a scaffold degrades faster than enough production of bone extracellular matrix can happen, cells will lose key physiochemical factors for bone tissue regeneration and repair, leading to scar formation. If residence time is prolonged, bone extracellular matrix production and cell proliferation will be suppressed [31].

Degradation rates are supposed by design to match the rate of new tissue development and maturation after transplantation in vivo [34]. The structure and microenvironment of scaffolds should ensure an increase in cell population, particularly for osteoblasts, which have bone-forming properties [43], leading to differentiation of precursor cells into osteoblasts and being supportive towards osteoblasts regenerative actions [8]. 

**Figure 3:**
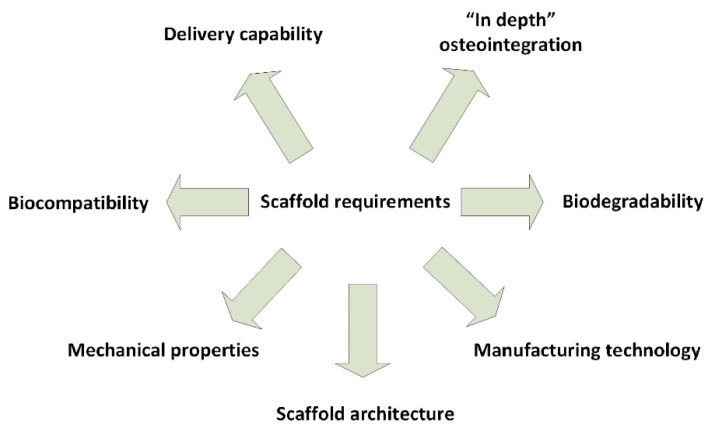
The key considerations for designing a scaffold or determining its effectiveness. Realized based on [24].

The key considerations for designing a scaffold or determining its effectiveness are as seen also in Figure 3 [24]:

1. Biocompatibility: it must trigger a reduced immune reaction to hinder it from causing a severe inflammatory response that might reduce healing or cause rejection by the body. Cells must adhere, function normally, and migrate onto the surface and eventually through the scaffold and begin to proliferate before laying down new matrix.
2. Biodegradability: it must degrade while new tissue is forming, and cells deposit their extracellular matrix. Secondary degradation products must be of low or absent toxicity and able to exit the body easily.
3. Mechanical properties: it should possess enough mechanical integrity to operate from implantation to the completion of the remodeling process. A balance between mechanical properties and enough porous architecture to allow cell infiltration and vascularization is essential.
4. Scaffold architecture: It should possess a structure of interconnected pores with high porosity to ensure cellular penetration and adequate diffusion of nutrients to cells within the construct and to the extra-cellular matrix formed by these cells. A porous interconnected structure is also required to allow diffusion of waste products out of the scaffold, and the products of scaffold degradation should be able to exit the body without interference with other organs and surrounding tissues. A critical range of pore sizes exists, for any scaffold, which may vary depending on the cell type used and tissue being engineered.
5. Manufacturing technology: It must be financially profitable and feasible to scale-up from research laboratory production to batch production, and thus be clinically and commercially viable. The progress to scalable production needs to comply with good manufacturing practice (GMP) standards for ensuring successful translation to the clinic and determining the way in which the scaffolds will be delivered and made available to the clinician.
6. Delivery capability: It is essential to mention that delivery capability through the releasing biological active agents can induce important properties such as faster healing [44,45], antimicrobial [46,47,48], and antitumoral activity [49,50].
7. “In depth” osteointegration: This requirement is strongly correlated with the pore size and porosity but also with the nature of the biomaterial. Bone grafting went through progressive improvement from the use of first-generation bone grafts (metals and alloys, i.e., titanium and its alloys, stainless steel, Co–Cr alloys) to second-generation bone grafts (ceramics and polymers, i.e., calcium phosphates, Al_2_O_3_, ZrO_2_; collagen, gelatin, chitosan, chitin, alginate, PLLA, PLGA), third-generation bone grafts (acellular composites or nanocomposites) and fourth-generation bone grafts (composites or nanocomposites containing cells or derived). The fifth generation in bone grafting is assumed to be “material design”, the additive manufacturing methods, along with some classical ones, being used in generating desired morphology to assure deep cell penetration inside the graft as well as to allow a controlled delivery of the biological active agents’ activity [50].

Plenty of material types have displayed osteoinductive properties, including natural and synthetic ceramics, especially calcium phosphate-based materials in various forms [9,12]. There is evidence for the osteoinductivity of calcium phosphate-based materials namely in the form of cements, coatings, sintered ceramics, and coral-derived ceramics in numerous animal models, while other ceramics, such as bioglass and alumina ceramic, have been lately confirmed to be osteoconductive [12]. 

Biomaterials effectiveness is assessed in animal metaphyseal osteoporotic models, the ones of critical size being the most clinically relevant ones, as healing occur only with the aid of a graft [51]. In clinical practice, unlike standardized animal models, every fracture is distinct [22]. As clinical studies regarding biomaterials are scarce, due to their classification as medical devices and the strict safety regulations derived from this, clinical data is insufficient to assess performance and research is ongoing [52]. 

Bioceramics are the basis for porous scaffolds (e.g., bone regeneration), solid scaffolds (e.g., reconstruction of ear ossicles), and powders (e.g., bone filling). Considering the way they interact with the host, bioceramics are classified into resorbable (e.g., some calcium phosphates, calcium sulphate, etc.), bioactive (e.g., bioactive glass), and nearly inert (e.g., zirconia). Bioactive and resorbable ceramics, are considered promising regenerative biomaterials and have been subject of clinical trials [4].

Calcium phosphates ceramics have high potential for bone regeneration applications, due to their injectability, bioactivity, biocompatibility, and ability to deliver stem cells, growth factors and drugs, including anti-resorptive (anti-osteoporotic) drugs [53]. Calcium phosphates have a crystalline structure and chemical composition resembling bone minerals. The widely used hydroxyapatite (HA), having the chemical formula (Ca_10_[PO_4_]_6_[OH]_2_), has a hexagonal structure [27], similar to apatite, the main mineral found in bone, but a low resorption rate [4], being thermodynamically stable at physiological pH and less soluble among other calcium phosphates [35]. HA scaffolds are well-known for being biocompatible, osteoconductive, and osteoinductive and other calcium phosphates, with faster resorption rates, such as β-tricalcium phosphate, have been studied as well [4]. 

Calcium phosphate scaffolds show the capacity to promote osteogenesis and osteointegration, due to their surface charge, and, also, their chemistry and topography [4]. HA coated implants are proven to promote osteointegration capacity of inert metals [7] but some pioneering works were reported related to COLL/HA composite deposition by using MAPLE [54]. Due to its high biocompatibility, bioactivity, osteoconductive and/or osteoinductive capacity (in certain conditions), nontoxicity, nonimmunogenic properties, and noninflammatory behavior, HA is available and used as a bone filler and as coatings on prostheses, [4]. 

Calcium phosphate cements also have applicability in osteoporotic fractures in the form of bone void fillers. HA is highly osteoconductive but has no natural osteoinductive capacity and incorporation of cells, growth factors, and also of various ionic substitutions into the crystal lattice such as Mg, Zn, F, Cl, Si, Sr, may prove to be beneficial [7]. The in vivo evaluation of scaffolds, loaded with growth factors and without them, indicated superior new bone development in previous variant compared to the other [4]. Therefore, a more efficient HA scaffold for osteoporotic applications can be made by the addition of bone regulatory agents/biologics with osteogenic and anti-resorptive capacities [7]. There is evidence that the body’s hard tissues contain substituting ions such as strontium (Sr) and fluorine (F) and incorporating them into HA can lead to improved material properties such as crystallinity and dissolution rate in physiological conditions [55] and various ions (Ca, Sr, Mg, Co, Cu, and Zn) can influence the implant generated immune response through the effect on immune cells and their cytokine releasing activity [21]. Bone formation is dependent on the extracellular microenvironment and its calcium ion gradient, both influenced by implant proprieties. Calcium ions dynamic and their concentration affect the recruitment and differentiation of mesenchymal stem cells (MSCs). Calcium phosphates ceramic adsorb firmly proteins from bodily fluids which further affect cell behavior and osteogenesis [56].

Natural marine sources for calcium phosphates are fish bones, seashells, corals, and algae. Phylum Porifera (Sponges) and Phylum Cnidaria (Corals) attracted attention due to their skeleton composed of bioceramics of which HA has been obtained. In addition, eggshells are an easily obtained, low-cost source for this material since they are considered food waste and calcium phosphates can be readily obtained by treating with phosphate, calcination and milling [4].

**Figure 4:**
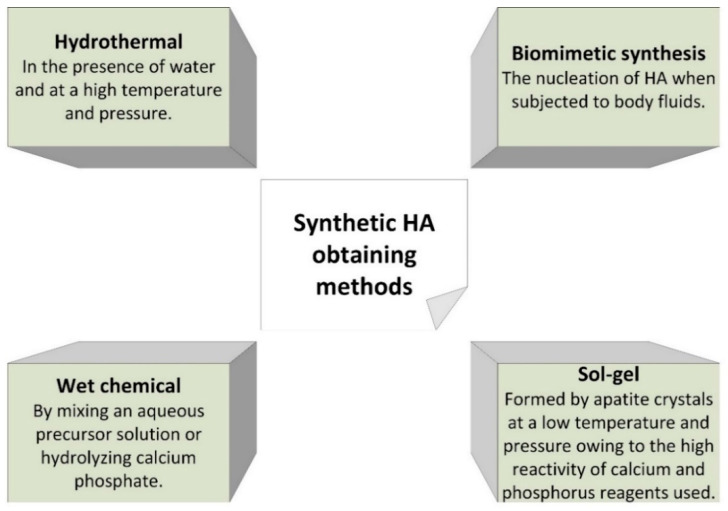
Synthetic HA obtaining methods. Realized based on [4].

Synthetic HA can be obtained by different methods, as seen in Figure 4. Parameters such as temperature, pH, reaction time, and concentration of reagents during synthesis can affect the properties of the obtained HA [4].

As the biological HA is formed from plate-like crystals with nanoscale thickness, it is considered that nanoparticles of synthetic HA such as bone apatite is the adequate choice to utilize for bone substitution and regeneration. Particle diminution to nano-size level may hinder demineralization. Nanoscale HA have advanced densification and sintering properties as a result of its increased surface energy preventing micro-cracks. Improved surface functional features in HA nanoparticles, compared to micro-size particles, may determine superior cell proliferation and differentiation [55]. 

However, the drawbacks for this material are still the brittleness, their high deformation rates [19] and lack a fine-tuning biodegrading capacity [4].

Natural polymers are expected to be suitable biomaterial scaffolds for bone tissue engineering, the most common being collagen/gelatin, chitosan, alginate, silk, hyaluronic acid, and peptides [9], the benefits being their capacity to resorb in vivo and their natural biocompatibility and reduced adverse immunological effects [2]. 

Synthetic polymers widely used for tissue engineering are aliphatic polyesters such as polyglycolic acid, polylactic acid, and polycaprolactone [9] and their copolymers [57]. Synthetic polymers scaffolds possess the advantages of versatile fabrication with a broad range of degradation rates and mechanical properties in contrast with natural polymers due to their composition, the copolymer ratio, and the interactions of their polymeric side chains. The options regarding chemical composition and 3D configuration can influence its cell adhesion capacity to incorporate and deliver bioactive molecules in a controlled manner [2]. They are used for scaffolds due to their good biocompatibility and suitability as drug delivery materials for desired molecules in tissue regeneration [31]. These polymers are already used in orthopedic applications for low mechanically loaded implantable devices such as screws and plates for fixing fractured bone fragments, but widespread use is still limited by their low mechanical performances [58].

PGA is highly hydrophilic, and its mechanical strength weakens in two to four weeks after implantation. PLA is more hydrophobic, and degradation can take from months to years after implantation. The degradation rates of these materials can be customized by copolymer blends (PLGA) utilization. Acid-catalyzed hydrolysis and large erosion of these scaffolds is a drawback, which can lead to structural instability and even stopped regeneration [31]. The PLGA scaffolds degradation rate varies, as the ones with wider pore size and reduced porosity degrade faster compared to those with reduced pore size and increased porosity due to higher surface area in the scaffolds with higher pore size, which increase the diffusion of acidic degradation products during the incubation period and lead to accentuated acid-catalyzed hydrolysis [42]. 

**Figure 5:**
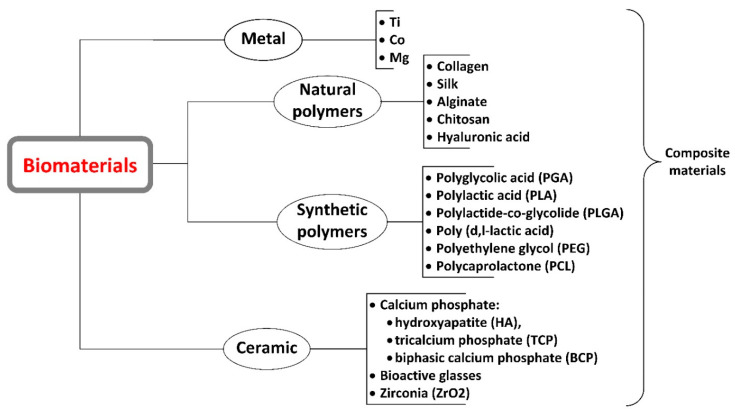
Different biomaterials for bone defect treatment. Realized based on [19,59].

Long-term exposure to PLGA acidic by-products upon degradation may cause tissue necrosis and implant failure. An array of polyphosphazenes has been incorporated in PLGA scaffolds, in order to control degradation rates and create more efficient scaffolds. Polyphosphazenes have nontoxic and neutral pH or basic products upon degradation and can be fine-tuned according to the degradation rates aimed [9]. pH is an important factor to take in account as the calcium phosphate precipitates start appearing with a rise in the pH and the sequential cross linkage of collagen chains depends on pH for bone regeneration [35]. The majority of polymer materials do not have osteoconductive properties and can lead to stress relaxation behavior and creep [16]. Figure 5 present some of the most important biomaterials used in bone tissue engineering: metals and alloys, polymers, and ceramics, but also derived composite materials. 

Polymer-ceramic composites represent attractive options for bone substitution and regeneration as it is hard for a single category of biomaterial to fulfill all the requirements of artificial bone scaffolds [27] and fully replicate the properties of bone [59]. Composites take advantage of each of its constituents, organic and inorganic (i.e., biodegradable polymer and ceramic materials), and prove to be more successfully strategy than the separate use of these materials [12]. In fact, this approach is a biomimetic approach which compositionally mimic the nature of the bone, which is also a polymer-ceramic composite material.

Scaffolds fabricated from polymer-ceramic composites biomaterials possess the higher biocompatibility and biodegradability of organic materials and the higher mechanical properties and biological activity of inorganic materials [27]. Because natural bone structure is, indeed, that of a composite biomaterial with an inorganic component—HA crystals and an organic component—collagen fibers [12], the combination of those two components for scaffold material can reproduce the chemical composition of the genuine bone extracellular matrix [27]. Evaluation of scaffolds is generally devised through evaluation of cellular interactions, the scaffold microstructure and mechanical properties [4].

The scaffold microstructure (e.g., pore size, shape, and interconnectivity) allows cell migration, nutrient and waste interchange, angiogenesis, and bone ingrowth and mechanical features ensure the formation of bone tissue after implantation while maintaining its structural integrity [4,9], while porous structures improve mechanical performance [4]. The mechanism of bone fixation is through the deposition of a surface layer of apatite, a process known as bioactivity of biomaterials [60]. This deposition arises from the merging of calcium derived from the bioactive material matrix or the biological fluids and phosphate present in the biological fluids [35]. Physiological fluids such as blood are supersaturated with these ions, but the deposition of the apatite layer is not happening on every surface. In vitro physicochemical analyses showed quite the opposite as faster deposition of apatite layer occurs on more reactive surfaces [16].

Polymer-HA composites containing PLA, PLGA, collagen, gelatin, and chitosan have been realized and proved to be osteoinductive, enhancing bone development in vitro and/or in vivo [12] and ectopically [9]. Combining HA and natural polymer has been proved to enhance mechanical properties and bioactivity of scaffolds in contrast to simple polymer scaffolds and to increase hydrophilicity, in case of PLLA-matrix [58], and potentially reduces adverse effects resulting from degradation of various synthetic polymers, [12] thus making polymer-ceramic composite biomaterial the most promising option for bone scaffolds [27]. 

A scaffold combining collagen, calcium phosphate, and HA, showed higher mechanical strength compared to scaffolds fabricated only from calcium phosphate, with proper biocompatibility, increased osteoinductivity and bone formation in vivo compared with calcium phosphate scaffolds and collagen-calcium phosphate scaffolds [4]. HA is one of the most promising fillers for the reinforcement of PLLA due to its high hardness and Young’s modulus [58].

These biomimetic materials are capable of simulating the formation, precipitation and deposition of apatite from simulated body fluid (SBF), leading to increased bone-matrix interface strength through better osteoblastic cell survival, proliferation and expression of bone-specific markers (i.e., bone sialoprotein and osteocalcin) [12]. 

Surface modification and functionalization of scaffolds with biologically active molecules can increase the effectiveness of bone regeneration. A specific molecule (i.e., peptide) chosen to initiate attachment of cells and their proliferation or directly their differentiation, can be deposited on scaffold surface or integrated within [8]. 

To overcome the challenge caused by the relatively low compatibility at the interface between bioceramic and biopolymer, the use of coupling agents has been proposed in the form of a chemical moderating agent with two different functional groups, the first being able to react with polymer molecules, while the second being able to adsorb with the ceramic surface to create a strong bond. There is substantial difference in chemical and physical properties between the two phases, which results in poor bonding strength. Interface interaction are important in determining mechanical properties that need more consideration, as research focused mainly on the reinforcement effect that bioceramics have on biopolymer matrix. The use of a coupling agent can enhance the efficiency of interfacial stress transfer between the two components, thereby improving the mechanical properties of the composite scaffold [27].

As a result of the different properties of bioceramic and biopolymer, it is improbable for bioceramics to disperse equally in the organic component and their low compatibility and interface bonding strength may lead to shrinking and deformation of the polymer on the ceramic surface and cause micro-cracks at their interface throughout processing, further leading to depreciated mechanical properties. After transplantation into the human body of a composite scaffold, the interface layer between the two components is rapidly damaged, and the ceramic is separated soon from the matrix, causing decreased mechanical property in the preliminary usage stage, unsuitable for a successful repairing process. In order to construct a successful bioceramic/biopolymer composite and use it as a bone scaffold is necessary to improve adhesion at the bioceramic and biopolymer interface [27].

## 5. Material Design by 3D Printing Technology

**Figure 6:**
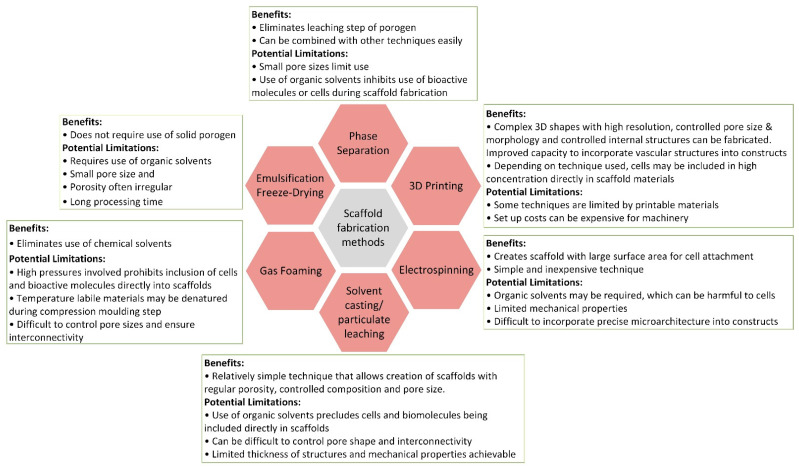
Benefits and potential limitations of different scaffold fabrication methods. Realized based on [59].

An innovative area of research concerning scaffold designing and fabrication is the use of additive manufacturing technologies (3D printing) [8] which guides the path in developing 3D structures trough cutting edge methods utilizing various biomaterials [61]. Advances in this field of research are providing the methods to produce complex and highly specialized 3D structures [31] serving as templates in providing a suitable environment for bone tissue regeneration [9]. This is why additive manufacturing techniques open new opportunities and there are premises to consider a fifth revolution in bone grafting materials due to the advanced “materials design”. Three-dimensional (3D) printing is realized through adding materials to obtain scaffolds using 3D model data, layer-by-layer, in contrast to subtractive manufacturing methods [62]. These methods have a great flexibility in producing scaffolds of different structural complexity [2]. A concise comparison between different scaffold fabrication methods can be seen in Figure 6. 

The 3D printed scaffolds are advantageous because of their customized shape, porosity/pore size, and mechanical characteristics [62]. This high customization permits a high control regarding the scaffold architecture, flexibility in scaling up fabrication, technically precise reproducibility—which is not always the case in subtractive fabrication techniques [2]—and a precise morphology [4]. The usage of 3D printing for bone tissue engineering possesses an essential advantage, such as the increased spatial control over the deposition of cells and materials that permits design control of complex tissue interfaces [13]. The capacity to precisely shape mechanical and biological properties, and degradation kinetics is linked with control of the scaffold architecture. Additionally, these methods allow the development of dimensionally precise prototypes of bone [4]. The development of 3D printing through computer-aided design (CAD) modelling further increases manufacturing precision and repeatability of scaffolds, and highly controlled porosity at both micro and macro levels [59]. Relevant to mention, is that the macroporosity is important in allowing for a fast cell penetration, fast bone ingrowth, but also in allowing vascularization oxygen and nutrients exchange, etc., while the microporosity is essential in designing the release profile, for instance.

Fused deposition modeling (FDM) 3D printing technology allows printing with polymer and polymer-based composite filaments by extrusion with a resolution of up to 200 microns. Such high printing precision allows designing and manufacturing with high accuracy personified highly porous scaffolds possessing an interconnected system of open pores, thus prompting osteointegration of scaffold [58]. 

Suitable polyesters for 3D printing of polymer scaffolds, possessing biodegradable and biocompatible properties, like poly(L-lactic acid) (PLLA), poly(vinyl alcohol) (PVA), poly-β-hydroxybutyrate (PHB), polyurethane elastomers, poly(3- hydroxybutyrate-co-3-hydroxyvalerate) (PHBV), poly(D,L-lactic acid) (PDLLA), poly(lactic-co-glycolic acid) (PLGA) and polycaprolactone (PCL) and polyurethanes, can be processed into wires, pellets and powders with the help of high temperature melting-extrusion and sintering, or by dissolution in organic/aqueous solvents to allow micro extrusion-based 3D printing at room/low temperature [62].

Microporous PLGA scaffolds were produced through rapid printing technique by precisely directing solvent streams onto polymer granules or by drilling with dies of a specific size. Reproducibility precision of these cylindrical scaffolds went to millimeter dimensions and the materials supported in vivo bone regeneration in defect models [31]. Due to its properties of thermoplasticity and extremely low thermal shrinkage, 3D printing technologies have also become widespread in PLLA molding [58]. 

Scaffolds obtained by 3D printing techniques represent an efficient approach for providing local delivery and sustained release of drugs, by mixing them with the synthetic/natural polymer solution, which can be 3D printed into scaffolds [62]. 

3D methods have precise control over scaffold microarchitecture and spatial content. Together with the wide variety of available bioactive materials, growth factors, functionalization techniques and biomimetic designs, increases the possibility to create complex scaffolds fine-tuned to individual needs, offering chances of treatment to difficult conditions, such as osteonecorosis, critical defects, and osteoporosis [59].

3D printing is a complex process with multiple engineering aspects to consider, due to the material type and 3D structure fabrication method when choosing the most suitable for specific damage or defect [61] and due to facing competition from injectable scaffolds in the form of hydrogels, micro or nanospheres [8]. In certain situations, when direct printing is problematic, such as for instance for the direct printing of the COLL/HA composite gel, the use of a precursor gel based on collagen and calcium hydroxide can be used and the printed microstructured patterns are simultaneously cross-linked with glutaraldehyde and calcium hydroxide are transformed into hydroxyapatite, both processes occurring as a consequence of dipping them into glutaraldehyde solution in phosphate buffer saline or SBF [41].

The following challenges are expected to be overcome in the near future [62]:

1. Imitating exactly the structure of natural bone tissue, as the majority of extrusion-based 3D printed structures have a reduced printing resolution and are capable of imitating the hierarchical structure at a rather reduced level. Superior micro-extrusion nozzle is expected to be capable of producing considerably higher resolution scaffolds;
2. Enabling the manufacturing of customized structures with complex characteristics, adapted to the heterogeneousness and gradient mechanical properties of defected bone tissue;
3. Controlled delivery of angiogenic factors and development of vascular-like duct in the structures are necessary to allow enhanced vascularization able to transport sufficient oxygen/nutrient to the regeneration site;
4. Obtaining both scaffold fabrication and cell incorporation concomitant, as 3D printed scaffolds loaded with cells are considered more effective in healing bone conditions, compared to recruiting host cells into scaffolds after implantation. Subsequent adhesion of cells on scaffolds in vivo usually induces variable cell distribution and low cell density, compared to in situ incorporation of cells through 3D printing, which is preferable. Besides 3D bioprinting, which fabricates cell-loaded hydrogel structure, no other 3D printing technique can sustain cell incorporation during the process;
5. Incorporating anti-bacterial or anti-cancer capabilities in order to treat infection/bone tumor resection-induced defects.

All these challenges can be considered part of the fifth generation of materials which will be obtained by “material design” and developed by using additive manufacturing.

## 6. Drug Delivery

**Figure 7:**
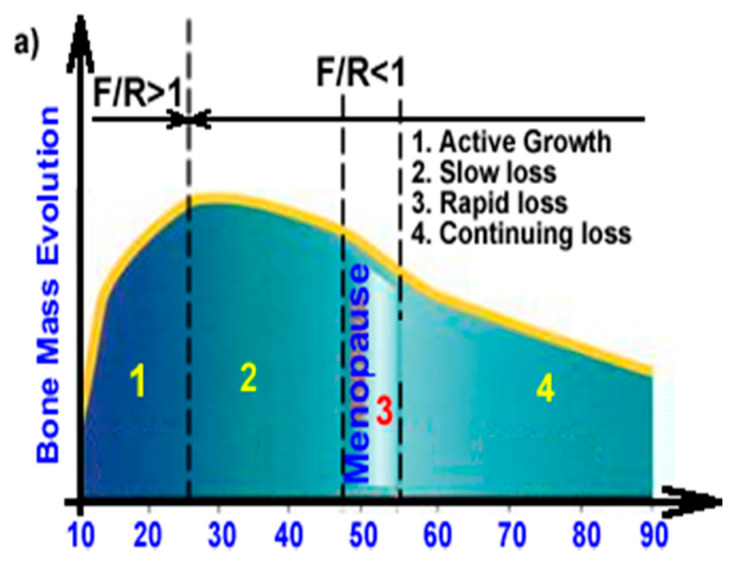

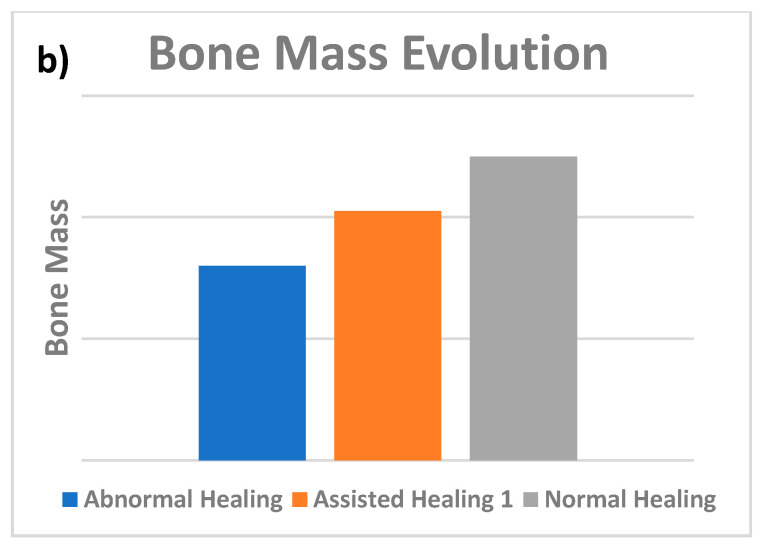
(**a**) Bone mass evolution during life; (**b**) bone mass evolution in normal versus assisted healing of bone defects.

Scaffolds made from biomaterials loaded with biologically active therapeutic agents (ions, vitamins, drugs) may become the preferable manner in which delivery of these agents at the defect site is realized in order to promote healing and integration [7]. Scaffolds act as reservoirs for delivery of bioactive agents including ions, such as, especially Ca^2+^, Zn^2+^, PO_4_^3−^; peptides and proteins, vitamins, genes but even cells and nanoparticles [3], aiding functional restoration of the fractured bone or to treat different diseases, especially infections, osteoporosis, or cancer [7] and their loco-regional delivery in order to minimize side effects at systemic level [41]. In case of osteoporotic fractures, convenient local drug delivery approaches are implant coatings, injectable bone cements and gels, while scaffolds are preferred for large bone defects [22]. The use of these biological active agents is especially welcome, as seen in Figure 7, because they can replace the normal bone resorption/formation ratio by enhancing the formation rate. Figure 7a represents the evolution of bone mass, highlighting the active growth period (formation rate (F) > resorption rate (R)) while, after 25 to 30 years the bone mass usually slightly starts to decrease, and this decrease will be more important after 45 years, especially if this is overlapping with menopause. Once the mass loss is starting, an important imbalance appears between formation and resorption and, in the case of fractures, the healing process will be significantly affected, and the bone density will be lower compared to normal, healthy bone tissue. Figure 7b schematically highlights the influence of the use of biological active agents in the healing of the osteoporotic, fractured bone. Therefore, especially in the case of fractures associated with osteoporosis, the healing could be slow, and the mechanical performances of these regions will be inferior and subsequently, secondary fractures can appear. 

The incorporation of several types of ions in the chemical composition of the scaffold is proved to promote the osteoconductivity of CPC [53]. HA can be an outstanding drug delivery carrier for numerous therapeutic agents as a result of its high stability, bioactivity, biocompatibility, and lack of toxicity [63]. In addition, biodegradable polymers have wide usage as drug delivery systems due to their biodegradability and biocompatibility, and, among them, aliphatic polyester (PLA), proteins (collagen, gelatin) or polysaccharides (alginate, chitosan) have the ability to extend the releasing of drugs from some days till some months [57,64,65,66].

The absence of an exothermic effect in the setting reaction and the natural porosity of CPCs permit the incorporation of therapeutic agents with reduced risk of thermal denaturalization and possible activity loss during preparation or implantation. Incorporation of drugs is realized by simply mixing it with one of the solid or liquid cement components. Drugs can also be added by adsorption onto the scaffold or incorporated into polymeric microfibers or microspheres prior to mixing with the cement paste. Features that impact loading and release of therapeutic agents include the microstructure, porosity and surface area of the cements, drug incorporation method, and the interaction between the agent and the pre-set matrix [53].

Ion substitution of HA is widely researched lately due to its capacity to allow simultaneously both the stabilization of bone defect sites and locally delivering therapeutic ions [63], thus improving bioactivity or mechanical properties of the graft [67]. Ionically modified CPCs (i.e., with Sr^2+^, (SiO_4_)^4−^, Zn^2+^, Mg^2+^) were investigated for their capability to influence bone modeling and remodeling processes [53], Sr^2+^ receiving attention for its particular prospects for stimulating new bone formation, hindering cell driven bone resorption and raising BMD, and thus having large acceptance and usage in systemic osteoporosis therapy [63]. 

Interest is rising regarding the potential of carbonate substituted HA as a way to improve the bioactivity of the composites, adhesion and differentiation of osteoblast cells, collagen and osteocalcin expression and mineral deposition [60]. During an evaluation of the apatite formation capacity of biomaterials in SBF, there were reported visible differences in calcium uptake, the thickness of the freshly precipitated apatite layer and a confirmation of higher degradation rate of carbonate HA [60].

Fluorine can enhance HA crystallization and mineralization during bone formation, as fluorine containing HA, Ca_10_(PO_4_)_6_(OH)_2−x_F_x_ is reported to manifest the lowest solubility and higher biocompatibility and bioactivity comparing with HA, leading to an increase in the differentiation and proliferation of osteoblasts, inducing, thus, bone regeneration [55].

## 7. Osteoporosis

Sustaining bone mass depends on bone remodeling processes, which imply the equilibrium between the activity of bone resorbing osteoclasts and bone forming osteoblasts. Unbalanced resorption activity leads to decreased bone mass, altered bone quality, and higher fracture risk in osteoporosis [68]. The same equilibrium of bone remodeling can be affected because of skeletal senescence, which alter the integrity and biomechanical properties of both types of bone tissue [5]. 

Osteoporosis is a chronic condition [69], characterized by decreasing bone mass and quality [6], making elderly patients, both men and women with the general increase in life expectancy, susceptible to osteoporotic fracture, also known as fragility fracture, due to low-energy trauma [5] and delayed [43] or impaired bone regeneration potential, as such fractures usually do not heal and increase the incidence of subsequent fractures by two to three times in the proximity of the site [7].

Osteoporotic bones have a low BMD [70], because of a low capacity of bone regeneration, excessive activation of osteoclasts [5] and the increased osteoclast induced bone turnover, leading to high crystallinity of the HA in the bone and lower level of acid phosphate [7]. These changes in the microarchitecture cause a reduction in both the cross-linking efficiency and tolerance of the bone towards mechanical stress [7] and increased risk of bone fractures [70]. Osteoporotic fractures are the visible effect of the micro-alterations that raise the susceptibility of bone to the applied load [5].

Surgery is the main treatment strategy, but poor results are obtained because of various biological and surgical factors, which makes it hard to achieve a stable fixation and the failure risk (refracturation) following surgery is significant [5,20]. Treating osteoporotic fractures is difficult because of the reduced healing capacity, which correlates for osteoporotic patients with a far higher (~50%) failure rate of implant fixation than against non-osteoporotic patients [67], making their implants prone to pull out and failure [7], as osseointegration is inhibited by osteoporosis [71]. Decreased bone mass [69], porous structure and low strength are linked to a reduced supporting ability of an implant [20]. 

Delayed regeneration potential is influenced by compositional difference in vitamin D, non-availability of growth factors, while estrogen is important in stimulating RANK ligand secretion, leading, thus, to osteoclasts activation and bone resorption. For men, testosterone is key for maintaining BMD. Decrease of the parathyroid hormone (PTH) determines reduced calcium absorption and, consequently, a reduction in BMD [7]. The bone fragility specific to osteoporosis subsequent to menopause is caused by an imbalance at the cellular level between exceeding bone resorption and lower bone formation, and from an increase in the rate of remodeling at the tissue level [72]. Although bone turnover is a normal physiological process involved in microfractures repairing, its acceleration determines bone loss [73].

Throughout the evolution of osteoporosis, osteocyte numbers per unit of bone area are constantly declining, and consequently trabecular thickness lowers and intracortical porosity heightens. These significant modifications in the matrix composition and structure cause deteriorated bone quality and lower resistance to mechanical loading [5]. Red marrow starts to accumulate adipose cells, turning into white marrow. Simultaneously with the beginning of osteoporosis, other changes were reported, namely, decreased number, proliferation and osteogenic differentiation potential of MSCs present in the bone marrow micro-environment [7].

**Figure 8:**
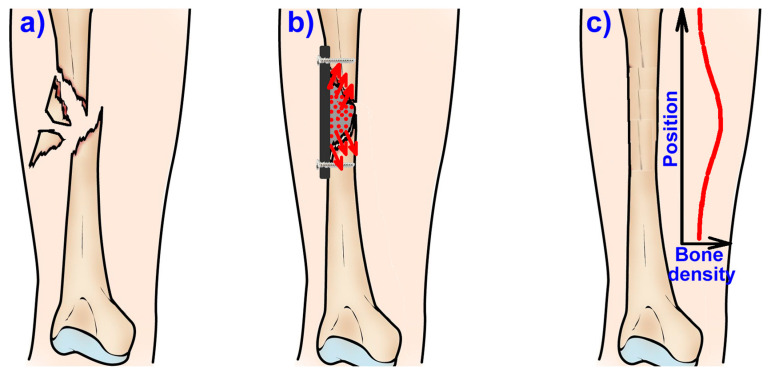
Schematic representation of bone grafting, healing and bone density distribution: (**a**) fracture; (**b**) bone grafting and healing; (**c**) bone density distribution.

Osteoporosis causes inadequate conditions for fracture healing [43], and the simple treatment of the acute fracture is unable to lower the risk of repeated fractures, thus, being necessary to be followed up by appropriate osteoporosis treatment [70]. In these cases, the loco-regional delivery could be an efficient solution because the delivered agents can counteract the bone density decrease and thus can assure an assisted healing where the formation rate is enhanced, the formation/resorption will be improved or even normal bone density can be obtained in the new bone as well as in the neighboring region where these agents are diffusing. In normal conditions, these biological active agents will diffuse during and after the healing process (Figure 8b,c) and the distribution of the bone density in this area will be similar with the one presented in Figure 8c. Similar approaches were also proposed in the patent application RO129822 (A2) [74] which use zinc as the active ion enhancing bone formation, but this approach can be applied also for developing anti-osteoporotic agents such as strontium ranelate. These drug delivery systems can be in solid or gel form, the solids will be implanted after surgical intervention as presented in Figure 8b and, if necessary, metallic supports can be used to take over the mechanical loadings while gels can be injected into the defect.

Osteoporosis is classified into primary and secondary [6]: 

Primary osteoporosis or postmenopausal osteoporosis appears after menopause. Type 1 affects more trabecular than cortical bone, as seen mainly in vertebral and distal radius fractures. It can affect men also, arising in midlife with vertebral fractures or low BMD by DXA. In men, genetic causes may involve genes for IGF-I18 or estrogen metabolism. Type 2 is found in both genders after age 70. Both types of bone are affected, commonly resulting in fractures of the proximal femur, in addition to vertebrae and radii. There are differences in aging-associated changes between genders, as women lose trabeculae and have greater spacing between trabeculae, and men only have thinning of trabeculae as they age. Quantitative computed tomography with finite element analysis have shown that women lose more cortical bone in vertebrae. Men have larger bones at peak bone mass; and with aging, more periosteal bone is deposited in long bones. These differences may explain why men fracture later in life than women [6].

Secondary osteoporosis affects both genders. The initiation of corticosteroid therapy is usually the cause of secondary osteoporosis, namely glucocorticoid-induced osteoporosis, which increase fracture risk as early as three months following oral glucocorticoid treatment [6].

Typical osteoporotic drugs act by either inhibiting bone resorption or favoring bone formation [7]. Currently, therapy for osteoporosis utilize anti-resorptive or anabolic drugs, which determine inconsistent effects on bone mass and fracture incidence [68]. Authorized treatment by FDA for osteoporosis includes the bisphosphonates (alendronate, risedronate and zoledronic acid), the more recent anti-resorptive antibody denosumab and the anabolic agent teriparatide. Bisphosphonates have been connected to bone osteonecrosis [71]. Ibandronate is also available, while strontium ranelate is approved to be used in over 70 countries but not approved by the FDA [6].

Bisphosphonates are a class of drugs that adjust the increased activity of the osteoclasts, and along selective estrogen receptor modulators (SERMs), calcitonin, denosumab and strontium ranelate, are included in the wider group of anti-resorbers. Differently from the catabolic drugs, anabolic drugs favor bone formation and include teriparatide and PTH analog [7]. 

Delivering a pair of drugs, with a complimentary action on bone dynamics has been considered, mostly with BPs and BMP-2—BPs and PTH being another combination. Simultaneous use of pro-anabolic and anti-catabolic drugs aims to aid impaired healing in osteoporotic patients [52].

New drugs against osteoporosis such as denosumab (targeting RANKL/RANK/OPG pathway) and romosozumab (targeting Wnt signaling cascades), are monitored for cardiovascular outcomes. One meta-analysis indicates that denosumab usage did not increase the risk of major adverse cardiovascular events among patients with primary osteoporosis over a period of 12 to 36 months but romosozumab might raise concerns [75], but after a re-analysis was performed, both Denosumab and Romosozumab association with cardiovascular outcomes was dismissed [76].

Romosozumab is a monoclonal antibody that inhibits sclerostin, an extracellular Wnt inhibitor secreted mainly by osteocytes [77], with a double effect of enhancing bone formation and diminishing bone resorption [75].

Clinical trials of romosozumab show that it is well tolerated and effective in fracture risk reduction, possessing great therapeutical potential for osteoporosis but still has some limitations by the fact that its anabolic potency is restricted to a few months of therapy, after which the large bone mass gaining is diminished. A possible strategy might be the usage of other drugs as well to sustain the bone mass gaining [78].

Odanacatib is an inhibitor of cathepsin K (CatK), and is very active, selective and sensitive, capable increasing bone formation and implant osseointegration and hinder bone loss in osteoporotic conditions. Osteoclasts perform bone degradation trough acid secretion in order to demineralize HA and lysosomal cysteine protease secretion (CatK) in order to degrade the bone matrix proteins. Odanacatib does not alter normal differentiation, migration and polarization of osteoclasts [71].

Increased risk of cardiovascular events, such as stroke, made odanacatib less attractive for future use against osteoporosis [75,79], even though it was proved beneficial for risk reductions of fractures in postmenopausal osteoporosis [75]. Other similar drugs such as balicatib, relacatib and dual cathepsin S/K inhibitor SAR114137 were discarded after adverse effects were observed [79].

Statins are not specific for treating osteoporosis but their capacity to inhibit the HMG-CoA reductase pathway determine an increased bone metabolism [22] and by increasing BMP-2 expression determine healing [52], but their efficiency is still debated. 

Clinical reports indicate intrinsic weak bone architecture and impaired osteointegrative ability as the lead cause of implant non-union and ejection. Worrying is also the negative effect osteoporosis has on previously osteointegrated implants [7], including degradable bone graft substitutes [43]. Implants used in osteoporotic fractures need to sustain, alongside mechanical support, a way to counteract the pathology of osteoporosis, thus, aiding bone regeneration. Consequently, local delivering of therapeutic agents that can increase osteointegration, osteogenesis while simultaneously regulate bone remodeling may prove advantageous [7].

## 8. Strontium Ranelate

Strontium (Sr) is an alkaline earth metal, present in the human body as a trace element, with chemical and physical properties comparable to those of calcium [23]. It is stored in the bone [33] where is stimulates cell growth, hindering bone resorption, opposing osteoporosis [55] and improving both cortical and trabecular bone structures [23]. It has been reported as a key factor in bone remodeling, being linked to both the stimulation of bone formation and hindering of bone resorption [80]. This beneficial double effect it has on bone turnover has been so far used to some extent against osteoporosis [43]. It has the capability to promote bone regeneration while reducing the frequency of fractures in animal models [23] even in small doses [43]. Sr ions have strong bone-seeking properties and often act in the human body similarly to calcium [69] but due to its higher atomic weight, incorporation of Sr ions reinforces the bone through increasing its mass and density [35]. Sr has attracted attention for potential use against osteoporosis as Sr ions increase osteoblast proliferation and reduce osteoclast activity, respectively [7]. 

Strontium ions have demonstrated effects both in vitro and in vivo studies [43], and is believed to lie in the Sr^2+^ capability to enhance osteoblast-related gene expression and the alkaline phosphatase (ALP) activity of mesenchymal stem cells (MSCs) [55], together with inhibiting the differentiation of osteoclasts [3]. ALP is considered a marker of osteogenic differentiation [28]. 

Sr hinders the formation of a ruffled border by the pre-osteoclast cells, thus hindering its maturation and resorption ability. Sr controls calcium sensing receptor activity by increasing calcium concentration in the microenvironment, resulting in an enhanced osteogenesis by osteoblast cells. Advanced osteogenic ability (ALP activity) of Sr doped CPCs is indicated by rat OB sarcoma cells model [7]. Sr doped HA/chitosan nanohybrid scaffolds display increased ALP activity, extracellular matrix mineralization, and osteoinductivity [63].

Sr ions promote protein synthesis for both collagen and non-collagen and osteoblastic growth [35] together with the inhibition of osteoclast differentiation and resorption activity, causing a denser and larger bone [23]. Considering this, Sr is believed to be effective in improving the properties of materials, especially bioactivity and biocompatibility, stimulating bone regeneration [80] and being effective against osteoporosis. Through increased expression of angiogenic factors such as VEGF and Ang-1, Sr promotes neovascularization [21]. 

Strontium ranelate is considered effective in fighting fragility fractures through improving the biomechanical properties of bone [5] reducing the risk of fractures in osteoporotic patients [69]. In contrast to other anti-osteoporotic drugs, Sr has a beneficial effect on the cortical bone, as its mass and thickness increase the following treatment with Sr in animal and clinical studies [23]. It was indicated to consequently reduce the chances of vertebral and hip fracture in elderly woman [35]. Sr has been proved effective in vitro in reducing osteoclast differentiation, activity and bone resorption and increasing osteoblast differentiation [70]. The metabolic activity of osteoblasts is enhanced in the presence of strontium ions, while the activity of the osteoclasts is reduced depending on the concentration of strontium in the microenvironment [43]. Inhibited activity of osteoclast in the presence of Sr is due to reduced synthesis of matrix metalloproteinase coupled with control of osteoprotegerin-RANKL pathway [81].

The mechanisms through which Sr can stimulate new bone formation are the CaR/ERK1/2 and Wnt signaling pathways. Wnt/β-catenin signaling is essential in the bone development and homeostasis. Wnt/β-catenin signaling is activated by Sr^2+^, which promotes new bone formation by inhibiting the osteoclasts and stimulating osteoblasts through nuclear factor NFATC1. Sr^2+^ concentration levels may lead to different results of new bone formation and side effects, meaning that accumulation of Sr^2+^ should be considered when applied to humans [33].

Cn-NFAT signaling is identified as a mechanism involved in strontium ranelate (SrRan) in vitro displayed osteoblastic stimulatory effects. This involves the activation of Cn-NFATc1 and components of canonical and non-canonical Wnt signaling, making this molecular mechanism the novelty through which SrRan enhanced osteoblastogenesis is explained [68].

Drugs containing strontium show significant effects in the prevention and treatment of osteoporosis [7]. Sr administered as SrRan is a known remedy against primary osteoporosis and difficult fracture healing, being approved for treatment many countries [43]. SrRan influences bone formation through a dual mechanism, by signaling osteoblasts to produce bone and inhibition of osteoclast mediated bone resorption [28]. SrRan in vitro results confirm its dual mechanism and in vivo was shown to determine an advantageous bone balance in experimental osteopenic models. In clinical usage it was also shown to decrease in patients with primary osteoporosis the risk of vertebral and non-vertebral fractures [68], femoral bone fractures also being positively impacted [63]. SrRan remains the main option for cases of severe osteoporosis [81]. 

SrRan is used for its gastric tolerability, physicochemical characteristics, bioavailability of Sr [82] and to decrease Sr side effects [83].

As SrRan rebalance bone remodeling by increasing bone formation and decreasing bone resorption [5,23,72], it is considered conducive to new bone formation and studies indicate a beneficial effect of SrRan on all parameters related to bone quality and strength [5]. Profiting of its both antiresorptive and anabolic properties, it can increase and stabilize BMD [70]. Its dual mechanism of action is favorable to osteoporotic fracture repair, in contrast with other drugs such as bisphosphonates, which have no effects on bone growth and repair of resorption sites of osteoporotic bone and can cause secondary effects such as inflammatory disease and osteonecrosis [28]. Osteoclasts might not be inhibited as in regular solely anti-resorptive treatment with bisphosphonates [43]. 

SrRan administrated orally in a daily dose of 2 g seems to be a safe approach and effective in decreasing the risk of vertebral fractures in postmenopausal osteoporosis patients [33,72] and documented to improve BMD, similar to other anti-osteoporotic drugs such as bisphosphonates, selective estrogen receptor modulators (SERMs), parathyroid hormone (PTH), and denosumab [23]. Among them, only Sr has a dual mechanism of action: stimulating new bone formation and reducing bone resorption [8,9], while increasing bone strength and decreasing fracture incidence [23]. Strontium ranelate increased BMD similar in both genders, both having fewer fractures after Sr administration [6]. Due to its anti-osteoporotic effect, ongoing treatment after the fracture occurs has a beneficial effect [23].

Despite all the shown benefits, systemic administration requires a high dose and prolonged drug administration period [7] and can lead to serious adverse effects in osteoporotic patients such as myocardial infarction, venous thromboembolism [43], and skin rashes [7]. The bioavailability of Sr via oral administration is about 25%, making local delivery with the aid of a biomaterial more effective [83]. The safety of SrRan has been questioned after the EMA found that its use increases the risk of heart problems in postmenopausal osteoporotic patients [84]. 

The Sr treatment is of particular concern because of its cardiovascular risk [84] and is restricted although it is still prescribed to patients who have no other suitable treatment [23], therefore the controlled local release of Sr is decisive as it has a great impact on biological processes [80]. It is not recommended in patients suffering the following conditions: peripheral vascular disease, ischemic heart, cerebrovascular disease, or uncontrolled hypertension [6]. Local release from scaffolds can prevent the side effects [43] as long-term administration is required for Sr to be effective. Research proved that local delivery at the injured site of a low dose of Sr is beneficial for osteoporotic bone healing and has clinical therapeutic importance [7]. In order to decrease the administered drug dose and bypass the adverse side effects, the design of a durable local SrRan delivery system would be clinically important [70]. The use of a low dose is not anticipated to induce persisting adverse side effects and delivering SrRan locally at the defect site through implanted scaffolds, may be a better strategy comparing to oral treatment [7]. 

In induced osteoporosis models of supercritical rat bone defects, Sr released from SrRan loaded bioactive glass, CPCs, calcium silicate and collagen sponges implanted had a visible accelerated bone regeneration effect depending on concentration [43]. Other osteoporotic models using rats and sheep proved the safety and efficacy of low dose Sr loaded implants (10%) [7]. Sr-containing CPCs has been described to improve bioactivity and biocompatibility, promote osteoblast proliferation and differentiation, and enable deposition of apatite, resulting an increased mechanical strength of the bone–scaffold interface, in both types of bone [80]. Chemical resemblance of Ca and Sr contribute in the development of Sr containing HA (SrHA) scaffolds [7]. 

Sr substituted calcium phosphates can impact in vitro the differentiation and activity of osteoclasts and osteoblasts [43] and SrHA demonstrates osteoinduction and high solubility, making the incorporation of Sr into HA beneficial for improving bioactivity [55]. 

Improved Sr and Ca release was noticed for SrHA in comparison to stoichiometric HA granules, which is expected to further healing in vivo through the anabolic and anti-catabolic properties of Sr [67]. When SrHA was added to an allograft it aided the healing of the area around the implant, a fact proved by the extended volume of new bone formed in the respective area, and delayed resorption [85]. Released Sr ions from the biomaterial should stimulate genesis of osteoblasts through ion release or indirectly by increased presence and activity of osteoclasts after material resorption, leading afterwards to osteoblasts stimulation [43]. 

SrHA coatings at Sr concentrations of 5%, 10%, and 20% showed increased implant osteointegration, improved trabecular microstructure around the implant, and better fixation based on the molar ratio of Sr ions. The results for Ti implants show that Sr could enhance new bone formation on implant surfaces, thus, improve osteointegration with the aid of SrHA and the 20% Sr loaded coating, and proved to be the most beneficial for implant osteointegration among the tested variant in osteoporotic rats [69].

Strontium was added into CPCs to augment their osteoconductivity and accelerate their degradation. Studies indicated that in vitro the rates of osteoblastic cell proliferation are enhanced and in vivo degradation is faster and osteoconductivity is better [53]. Beneficial effects of SrHA on bone mass at interface between implant and bone has been observed in healthy rabbit segmental bone defect model. Some sources indicate a daily dose of 2 mg/per body weight of SrRan in treating osteoporosis systemically [7].

Studies suggest that Sr loaded CPCs induce faster hydroxyapatite growth, high percentage in vitro cell viability and superior durability than the Sr free samples, in the case of Sr replacement of Ca, up to 5% in sol-gel bioactive ceramics with improvements on the proliferation rate of the cell line of human osteosarcoma [35]. It is much harder in vivo to trace and monitor the release, distribution, and accumulations of drugs. High concentrations can be monitored with magnetic resonance imaging (MRI) shortly after implantation, but postmortem examinations remain the most precise approach [83].

In the SrHA coating, Sr ions replace a part of Ca ions leading to an improved dissolution of the SrHA coating, thus, increasing the concentration of Sr, Ca, and phosphorus ions near implant surfaces. Their presence in the microenvironment improve osteoblast activity and new bone development into the implant surfaces [69]. While Sr can prove beneficial, high concentrations can have a negative impact [80]. Recommended concentrations range between 1% and 10% for osteoporotic applications. Using 10% SrHA scaffold to enhance in vitro osteoblast differentiation means a release of 0.11 mM Sr ions/mg is expected, enough to sustain a therapeutic effect in osteoporotic bone healing [7]. The reported minimum dose for in vitro conditions was 0.1 mM for osteoblast activation and 1 mM for osteoclast inhibition [83]. In addition, material properties such as strength, degradability, handling, and component release can be modified by loading with Sr compounds and these properties frequently have non-linear dependence on Sr concentration [43].

SrRan can be encapsulated, also, in PLA matrix using s/o/w and s/w1/o/w2 techniques, the latter being used in order to create sustainable SrRan delivery systems up to 24 wt% SrRan content, with observed precipitation of biomimetic calcium phosphate on the surface and in the pores of the delivery systems [70].

## 9. Conclusions

Osteointegration of implants and weakened regeneration potential of the host bone remain challenges to be overcome in osteoporotic fracture healing. Functionalized scaffolds capable of improving osteointegration and fast healing are of great importance for osteoporotic patients who have a lower number of osteoprogenitor cells. Loco-regional delivery of anti-resorptive and/or osteogenic factors at the defect site may have significant clinical applications for the recovery of fractured osteoporotic bones. Throughout this review, we have tried to resume the current state of research and the strategies for bone healing scaffolds and the future perspectives in the case of osteoporotic fractures by incorporation of biological active agents such as strontium ranelate in bioceramic/biopolymer composite bone scaffolds. The loco-regional delivery of SrRan can be suitable for administration because the systemic toxicity and especially the cardiovascular adverse effects can be reduced/removed but additional research will be necessary to evaluate the in vivo, short and long-term toxicity. According to the literature it is evident that, even if many researches were already done, many additional studies are necessary to reach optimal formulations. Along with the strontium-based formulations, some other biological active agents are usually used in the therapy of the osteoporosis and thus can be loaded in grafting materials to control the ratio between formation and resorption.
